# 

**Published:** 1895-08

**Authors:** 


### CONTENTS.

COMMUNICATIONS.

Tumors of the Mouth........ 365

PROCEEDINGS.

Twenty-Sixth Annual Session of the Alabama Dental Association, 1895........ 375
Mississippi Dental Association, 1895........ 383

MONTHLY DIGEST.

Operative Dentistry........ 392

SELECTIONS.

So Strange........ 374
A Death from Bromide of Ethyl........ 401
Phenacetin in Rheumatism........ 402
Report of the Collective Investigation Committee on Anæsthesia at the recent Surgical Congress in Berlin........ 402
Spasmodic Neuralgia of the Face........ 403
Ulyptol........ 416
Nitroglycerine........ 416

COMMENCEMENTS.

Western Dental College of Kansas City........ 404
University of Maryland—Dental Department........ 405
Birmingham Dental College........ 405
Western Reserve University—Dental Department........ 406
Royal College of Dental Surgeons of Ontario........ 406
Vanderbilt University—Dental Department........ 407
Meharry Medical College—Dental Department........ 407
University of Iowa—Dental Department........ 408
Columbian Dental College........ 408
Medical Department of the University of Denver........ 409
Ohio Medical University—Dental Department........ 409
National University—Dental Department........ 410
Columbian University--Dental Department........ 410
Cleveland University of Medicine and Surgery—Dental Department........ 411
The Boston Dental College........ 411
University of Michigan—Dental Department........ 412
Kansas City Dental College........ 412
University of Tennessee—Dental Department........ 413

EDITORIAL.

A Well Deserved Honor........ 414
Dr. Corydon L. Ford........ 414
Obituary—Dr. Augustus Woodruff Brown, D.D.S........ 415
Resolutions Adopted by the Odontological Society of Chicago in Reference to the Death of Dr. J. J. R. Patrick, of Belleville, Illinois........ 415

